# Conclusions of the expert panel: importance of erlotinib as a second-line therapeutic option

**DOI:** 10.1186/1753-6561-2-s2-s4

**Published:** 2008-09-24

**Authors:** Aldo Castagnari

**Affiliations:** 1 Centro Integral de Oncología. Leandro N. Alem 460 (1832) Lomas de Zamora, Provincia de Buenos Aires, Argentina

## Abstract

During the Experts Meeting on Lung Cancer, participants emphasized the usefulness of erlotinib as second-line therapy for lung cancer. They noted that, although there are no comparative studies, erlotinib could be as effective as docetaxel and pemetrexed in second-line therapy. Regarding the toxicity profile of each of these drugs – one of the key issues considered in the meeting – specialists pointed out how important it is to clearly identify existing differences in this issue. Each drug has different degrees of toxicity, and this information is crucial at the time of choosing the therapeutic regimen. Erlotinib treatment could be an effective option for second-line therapy.

## Effectiveness: erlotinib compared with docetaxel and pemetrexed

In this respect, the expert panel cannot give a conclusive answer because up to now no direct phase III comparative studies have been carried out on these drugs. By analyzing current evidence, they can indirectly infer that, for second-line treatment, erlotinib seems as effective as docetaxel in terms of survival. In spite of the lack of comparative experiences, pemetrexed would be at least as effective as docetaxel, and erlotinib might be as effective as the other two drugs.

In the first-line setting, cytotoxic chemotherapy is considered to be superior to erlotinib as regards overall patient population [1-3]; and in third-line therapy, erlotinib is the only effective treatment. However, in the second-line setting, a thorough selection of patients is required [4]. If it is concluded that these drugs are similar regarding efficacy, the decision should take into account individual aspects of each case. For instance, in a smoker without comorbidities, chemotherapy will probably be preferable. In contrast, in a female with adenocarcinoma and no smoking history, treatment with erlotinib seems to be the most suitable option, though according to the study review, erlotinib has been seen to be beneficial for all subgroups [5,6]. This clearly shows that on no account are these treatments competing, but that each case's particular aspects must be contemplated. For example, erlotinib could be considered in elderly patients [7] (i.e., older than 70) and in those patients with suboptimal performance status or comorbid conditions.

## Toxicity/safety of drugs indicated for second-line treatment of NSCLC

Experts have stressed the fact that by administering docetaxel and pemetrexed, hematologic and hepatic toxicity prevail, whereas with erlotinib the main toxicities are adverse skin and, to a lesser extent, gastrointestinal events. In fact, docetaxel and pemetrexed are associated with febrile neutropenia, while this complication has not been reported with erlotinib.

One of the reasons to discontinue erlotinib is its biological effect, seen in the occurrence of treatment-related rash. Patients with severe conditions who see their appearance seriously affected often refuse to continue taking medication. Faced with this situation, physicians should learn how to deal with skin effects and be well aware of toxicity grade; in case of toxicity grade 3, the dose should be reduced or the administration scheme modified, including treatment discontinuity for less than two weeks and then resumption of usual dose.

Evidence shows that second-line therapeutic options seem to be roughly comparable as regards response [8,9]. In order to choose the most convenient therapy, experts should evaluate the conditions in each case together with the patient, who should be well informed as to the toxicity profile of each drug. To stress the fact that the toxicity of each therapeutic option is different is essential.

Data seem to suggest that the safety profile of erlotinib is more favourable than that of commonly used second-line drugs. While in some cases the administration of erlotinib produces biological skin effects, these can be controlled by an adequate intervention on the part of the physician, who may adjust the dose or, in extreme cases, discontinue it temporarily.

A direct relationship between the skin effect and the therapeutic response to erlotinib has been observed [10-12]. The greater the skin toxicity, the better the survival seems to be. This may be a key observation to obtain consent from patients who will undergo treatment with erlotinib. It should be noted that while there are no reported cases of death related to erlotinib treatment, death has been reported with the use of the other drugs.

Experts have agreed on the importance of interdisciplinary work in the treatment of toxic effects. Multidisciplinary care allows comprehensive assistance and specific solutions for each individual area. This is essential to help patients experiencing serious adverse effects or effects that have a high impact on quality of life, since most often the resulting impairment is very hard to deal with. With erlotinib therapy, if toxic skin effects occur, the assistance of and joint work with skin specialists is recommended.

At this point, regarding the outcome of toxic skin effects, patients who develop significant rashes do so usually in the 2^nd ^or 3^rd ^week after treatment onset. Then, lesions usually begin to disappear few weeks later.

It is important to point out that erlotinib is administered orally and only once a day. This is a great advantage over other therapeutic agents requiring intravenous administration, which may have negative effects. In addition, unlike the other currently available options, erlotinib does not require the administration of pre-established additional medication.

## Patient selection

So far, evidence is inadequate to establish a clinical or molecular profile for patient selection. Many of the data come from observations in non-randomised trials. The greatest therapeutic benefits were reported in female non-smokers with bronchioloalveolar carcinoma.

Prevailing information about molecular profile was initially provided by clinical trials of gefitinib, where a higher response rate was observed among female non-smokers with adenocarcinoma. From then on, the idea has prevailed that the population with these characteristics is more likely to show a favourable response.

While certain similarities are seen with erlotinib, a great number of patients has been shown to have a favourable response (not necessarily a longer survival), despite their having different characteristics. Notwithstanding, the best clinical response in the second-line setting is apparently achieved in female non-smokers with adenocarcinoma.

At present, there is no evidence to select the candidate patient for erlotinib through the clinical or molecular profile, though there are patient subgroups that benefit more than others [13].

## Relationship between toxic skin effects and biological effect of erlotinib

The BR.21 [6] trial shows that the most important toxic effects of erlotinib are firstly related to skin rash and, secondly, to diarrhea. Regarding response prediction, literature on this subject points out that skin adverse effects would be a surrogate marker of the biological-therapeutic effect. Skin toxicity curves and their corresponding responses have been published: the higher the skin toxicity, the higher the response. Specifically, reports show that patients with grade 3 and 4 rash had an 80% response. In contrast, individuals with grade 2 toxicity showed a much lower response (12%). In this respect, all experts have agreed: rash may be an objective marker of response to erlotinib treatment [10-12].

## Conventional chemotherapy vs. targeted therapies (in the first-, second- and, if necessary, third-line setting)

Unlike other drugs, erlotinib has shown clinical activity in all therapeutic lines. Therefore, it would be the physician's decision to administer it, based on each patient's characteristics – and clinical considerations as well –, for example, taking into account the drug's availability. However, erlotinib is not currently accepted as a first-line treatment.

In third-line treatment, there are no doubts regarding the indication of erlotinib; in second-line, according to the opinions presented, there is none either. However, for the time being, scientific evidence is not enough for first-line treatment, and future results have to be awaited.

Special cases may arise, such as patients who are not candidates for or refuse chemotherapy and who are prescribed erlotinib as first treatment option. The use of erlotinib as first-line treatment – with some exceptions – will then depend on the results of ongoing studies. It should be noted that in previous phase III randomized trials, combination cytotoxic chemotherapy with erlotinib in the first-line setting was shown to be no more beneficial than combination chemotherapy alone. [2,14,15].

## Differences between HER1/EGFR inhibitors: erlotinib and gefitinib

There are no studies comparing these two drugs. Evidence suggests that erlotinib is better than gefitinib: the former had significant impact on survival rate, while the latter did not.

Anecdotal observations suggest that the grade of skin toxicity is much higher for erlotinib than for gefitinib. With gefitinib, grade 3 and 4 toxicity is generally low. For adverse gastrointestinal and skin events, similar data were reported; i.e., the incidence and grade of rash and diarrhea are higher with erlotinib than with gefitinib. This fact may explain the different therapeutic results obtained.

The pharmacokinetic explanation accounting for the difference between gefitinib and erlotinib lies in the fact that in the ISEL trial gefitinib was not dosed close to its maximum tolerated dose (MTD), a fact which is also related to the grade of adverse effects reported. If the optimum dose is lower than the MTD, adverse effects will then be lower since plasma concentrations are not the ideal ones and effectiveness is also lower. Whether higher doses of gefitinib are more effective, yet also more toxic, should be determined. Conversely, erlotinib is administered at an optimum dose that is very close to the MTD; thus, biological side effects are higher. Clinical evidence shows that erlotinib is more effective than gefitinib regarding impact on survival, with respect to placebo [16].

## Influence of pharmacokinetic (PK)-pharmacodynamic (PD) relationship of erlotinib on response and safety

There are a number of well-known recommendations regarding this subject, such as avoiding the intake of some citrus fruits and the combination of treatment with anticoagulants and antifungals, among other contraindicated drugs.

There are no practical recommendations regarding pharmacokinetics, except that it is important for the patient to take the medication under fasting conditions so as to preserve ideal plasma concentrations, and preferably not to smoke during treatment.

## Cost-effectiveness: erlotinib compared with other therapies

Overall, cost-effectiveness comparisons with the other two options for second-line treatment show higher benefits for erlotinib in international studies applied to other countries.

In Latin America, two studies analyzing cost-effectiveness took into account not only medication and health service costs but also the cost of the complications that may arise from each therapy administration, mainly chemotherapy-related febrile neutropenia and erlotinib-related rash and diarrhea [17]. According to research carried out with docetaxel and pemetrexed, which took into account therapy overall costs, including additional medication (e.g., colony-stimulating factor, antibiotics) and mainly hospitalization costs, the cost of docetaxel was higher as compared with that of pemetrexed.

There is no knowledge of cost-effectiveness analyses of erlotinib carried out by local health institutions. However, an independent consulting firm performed a local analysis focused on women with adenocarcinoma, and compared erlotinib's cost-effectiveness with that of docetaxel, showing a cost-effectiveness advantage for erlotinib. This analysis took into account response to treatment and patient survival based on local data from the TRUST trial, and official costs of hospital resources and supplies.

From these data, they might say that cost-effectiveness of erlotinib is comparable to the rest of therapies or, at least, is not higher. According to available data from cost-effectiveness analyses worldwide, docetaxel and pemetrexed are equivalent to erlotinib. Further specific local studies for each health institution will be required.

## Timely referral of patients with lung cancer

The education general practitioners receive on oncology questions is most important. Information should be spread to all the medical community that there are effective therapies for lung cancer, not only aimed at survival but also at quality of life, and that there are increasing options for first-, second- and third-line treatment.

Lung cancer is a disease that requires a multidisciplinary, multiinstitutional approach. The intervention of different secondary and tertiary care institutions is needed, as well as the implementation of better local epidemiologic systems. Lung cancer presentation and histopathology are varied, thus requiring joint work to improve patients' referral so that they may receive the most appropriate treatment.

## Competing interests

Dr. Aldo Castagnari declares that he has no competing interests.
